# Pembrolizumab-Induced Tubulointerstitial Nephritis in Early-Stage Triple-Negative Breast Cancer: A Case Report

**DOI:** 10.7759/cureus.98726

**Published:** 2025-12-08

**Authors:** Catarina Ferreira, Cristiana H Martins, Renata Carvalho, Roberto Silva, Jorge Rodrigues

**Affiliations:** 1 Medical Oncology, Braga Local Health Unit, Braga, PRT; 2 Nephrology, Braga Local Health Unit, Braga, PRT; 3 Pathology, Unilabs, Porto, PRT

**Keywords:** acute tubulointerstitial nephritis, early-stage triple-negative breast cancer, immune checkpoint inhibitors, immune-related adverse events, pembrolizumab

## Abstract

Immunotherapy has become an integral part of cancer treatment, with immune checkpoint inhibitors (ICIs) increasingly used across multiple tumor types, including early-stage triple-negative breast cancer. However, these therapies can trigger immune-related adverse events (irAEs) that may involve various organ systems. We report a case of acute tubulointerstitial nephritis (ATIN) secondary to pembrolizumab in a woman in her 50s treated for early-stage triple-negative breast cancer. After three cycles of neoadjuvant chemotherapy combined with pembrolizumab, the patient presented with acute kidney injury (AKI) and elevated inflammatory markers. Infectious, obstructive, and other secondary causes were excluded. A kidney biopsy was performed, with findings consistent with ATIN, most likely pembrolizumab-induced. Pembrolizumab was discontinued, and corticosteroid therapy (prednisolone 0.5 mg/kg/day) led to rapid improvement and near-complete recovery of renal function. This case highlights the importance of early recognition and prompt management of ICI-related AKI to prevent irreversible kidney damage.

## Introduction

Immunotherapy represents a significant advancement in the development of novel cancer treatments. Of the various immunotherapeutic approaches, immune checkpoint inhibitors (ICIs) have transformed the management of multiple malignancies, demonstrating substantial clinical benefits and improved patient outcomes in recent years [1]. In the KEYNOTE-522 trial, the addition of pembrolizumab, a humanized monoclonal antibody that targets programmed cell death protein 1 (PD-1) and exhibits antitumor properties, to neoadjuvant chemotherapy, followed by adjuvant pembrolizumab administration, demonstrated significant improvements in pathological complete response rates and overall survival among patients with early-stage triple-negative breast cancer, while maintaining an acceptable safety profile [2-4].

Based on these findings, the integration of pembrolizumab into chemotherapy regimens as first-line therapy in the neoadjuvant setting for early-stage triple-negative breast cancer has become standard practice. Despite its clinical benefit, the administration of pembrolizumab is associated with a broad range of immune-related adverse events (irAEs), most commonly affecting the gastrointestinal, dermatologic, and endocrine systems. Although renal toxicity is an uncommon side effect that was not identified in initial studies, it warrants clinical vigilance given its potential severity and risk of irreversibility. In fact, renal irAEs continue to be underreported, despite being clinically significant due to their potential severity and the risk associated with delayed diagnosis [1,5]. There is also limited research on ICI-related AKI in early-stage triple-negative breast cancer.

We present a case of ICI-related AKI, manifesting as acute tubulointerstitial nephritis (ATIN), in a female patient in her 50s with early-stage triple-negative breast cancer who received neoadjuvant chemotherapy in combination with pembrolizumab.

## Case presentation

A 50-year-old postmenopausal woman, with an Eastern Cooperative Oncology Group (ECOG) performance status of 0, history of depression (medicated with sertraline 50 mg per day) and with no family history of cancer, was diagnosed with stage II triple-negative left breast cancer (cT2N0M0, according to the TNM (tumor, node, metastasis) classification by American Joint Committee on Cancer, AJCC, 8th edition [6]). Neoadjuvant treatment with chemotherapy (paclitaxel: 80 mg/m^2^, every week; and carboplatin: dose based on an area under the concentration-time curve of 5 mg/mL/minute, every three weeks) plus pembrolizumab (200 mg, every three weeks) was started.

The first three cycles of the treatment were well tolerated. The fourth cycle was delayed by a week due to fever and respiratory infection symptoms, and the patient immediately started empirical antibiotics (amoxicillin 875 mg plus clavulanate acid 125 mg, every 12 hours). Five days later, she was presented to the emergency department owing to a lack of clinical improvement and abdominal pain. Physical examination was unremarkable.

Blood workup revealed anemia and mild leukocytosis with neutrophilia, as well as elevated serum creatinine (sCr) and markedly increased C-reactive protein (CRP). Urine sediment analysis showed leukocyturia with elevated albumin-to-creatinine (ACR) and protein-to-creatinine (PCR) ratios, without hematuria or eosinophilia. Table 1 summarizes laboratory results from admission.

**Table 1:** Laboratory findings from hospital admission. Reference ranges correspond to institutional laboratory standards.

Lab Parameter (units)	Patient Values	Reference Range
Blood workup		
Hemoglobin (g/dL)	10.3	12.0 – 16.0
Leukocytes (/µL)	12,100	4,500 – 11,000
Neutrophils (/µL)	9,300	2,500 – 7,000
Eosinophils (/µL)	100	0 – 500
Platelets (/µL)	435,000	150,000-400,000
Urea (mg/dL)	53	19-49
Serum creatinine (mg/dL)	1.5 (baseline 0.6)	0.6 – 1.1
C-reactive protein (mg/L)	240	< 0.5
Urine analysis		
Albumin-to-creatinine ratio (mg/g)	170	< 30
Protein-to-creatinine ratio (mg/mg)	1.1	< 0.2
Leukocytes (per field)	125	< 10
Eosinophilia	Absent	—
Hematuria	Absent	—
Granular or WBC casts	Absent	—

The respiratory virus test was negative for Influenza, SARS-CoV-2, and respiratory syncytial virus. Thoracic x-ray and computed tomography (CT) showed no alterations, as well as abdominal x-ray and ultrasound. Renovesical ultrasound demonstrated kidneys with normal morphology and good parenchymal-sinus differentiation, excluding calculi, hydronephrosis, and focal lesions. The patient was admitted to the Oncology ward for further investigation. All infectious studies, including respiratory virus, stool tests for adenovirus/rotavirus, parasites, C. difficile, fecal and urine cultures, were negative. Both central (via central venous catheter) and peripheral blood cultures, drawn twice, were also negative.

Despite intravenous hydration and empirical broad-spectrum antibiotics, her renal function continued to worsen (maximum sCr: 2.3 mg/dL), and the inflammatory parameters remained elevated (maximum CRP: 248 mg/L) within the next five days. She repeated a renovesical ultrasound and abdominal CT, exhibiting only increased kidney volume, without other alterations. Immunologic studies (antinuclear antibody (ANA), antineutrophil cytoplasmic antibodies (ANCA), and complement levels) also came back negative. Given the patient's clinical presentation and negative workup, amoxicillin/clavulanate was deemed an unlikely cause of ATIN, as were infectious or sepsis-induced AKI.

Following nephrologists’ evaluation and the exclusion of alternative etiologies, a diagnosis of interstitial nephritis secondary to pembrolizumab was suspected. The patient started corticosteroid treatment with oral prednisolone at 0.5 mg/kg per day (30 mg daily), and a kidney biopsy was performed.

The patient was discharged after one week following improvement in renal function (sCr: 1.4 mg/dL) and a reduction in inflammatory markers (CRP: 36 mg/L), with a corticosteroid taper scheduled over six weeks.

**Figure 1:**
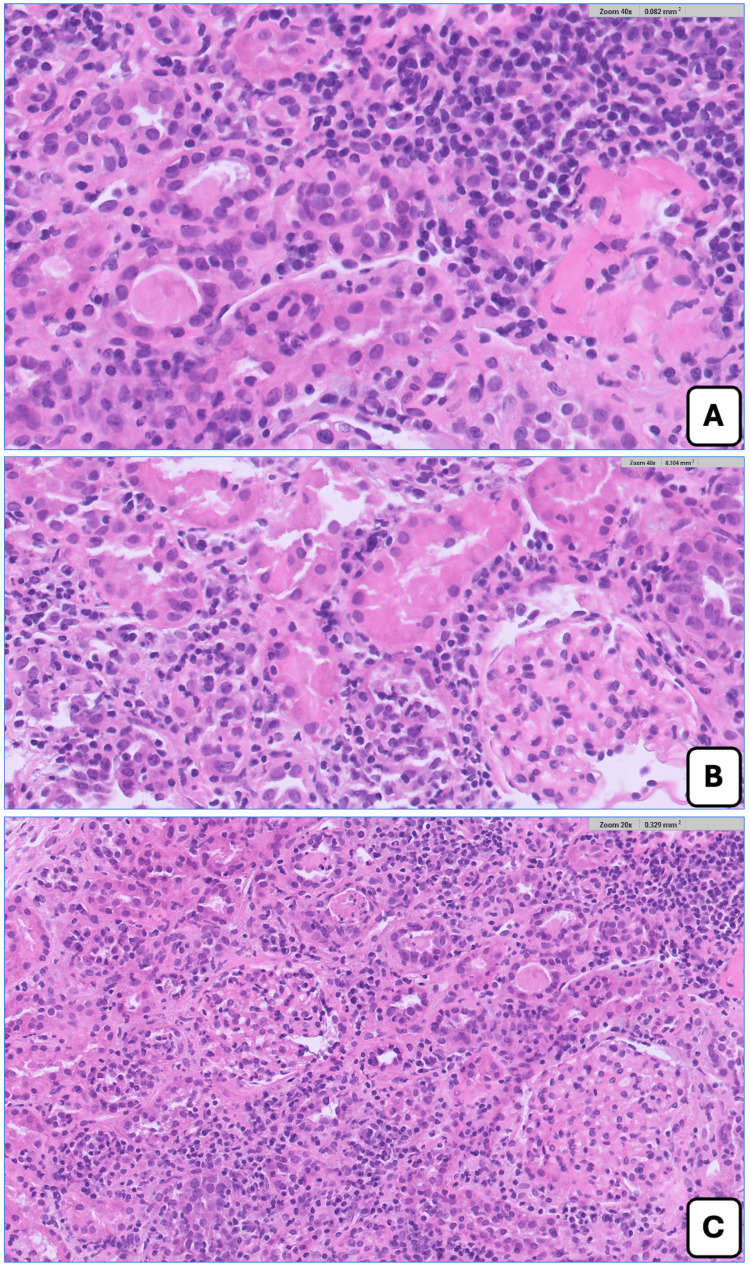
Renal biopsy findings (H&E stain). (A) Interstitial inflammatory infiltrate composed predominantly of lymphocytes with plasma cells and occasional eosinophils (H&E stain - 400x). (B) Evidence of tubulitis with inflammatory cells permeating the proximal tubular epithelium (H&E stain - 400x). (C) Low-power view showing widespread interstitial inflammation with preserved glomeruli and overall features consistent with acute tubulointerstitial nephritis (H&E stain - 200x).

In the meantime, renal biopsy showed preserved glomeruli and unremarkable vessels, with a moderate interstitial inflammatory infiltrate rich in lymphocytes, plasma cells, and occasional eosinophils, associated with tubulitis and acute tubular injury; mild interstitial fibrosis and tubular atrophy were present, and the immunofluorescence study was unremarkable (Figure 1). Overall, these findings were consistent with ATIN, most likely pembrolizumab-induced.

**Figure 2:**
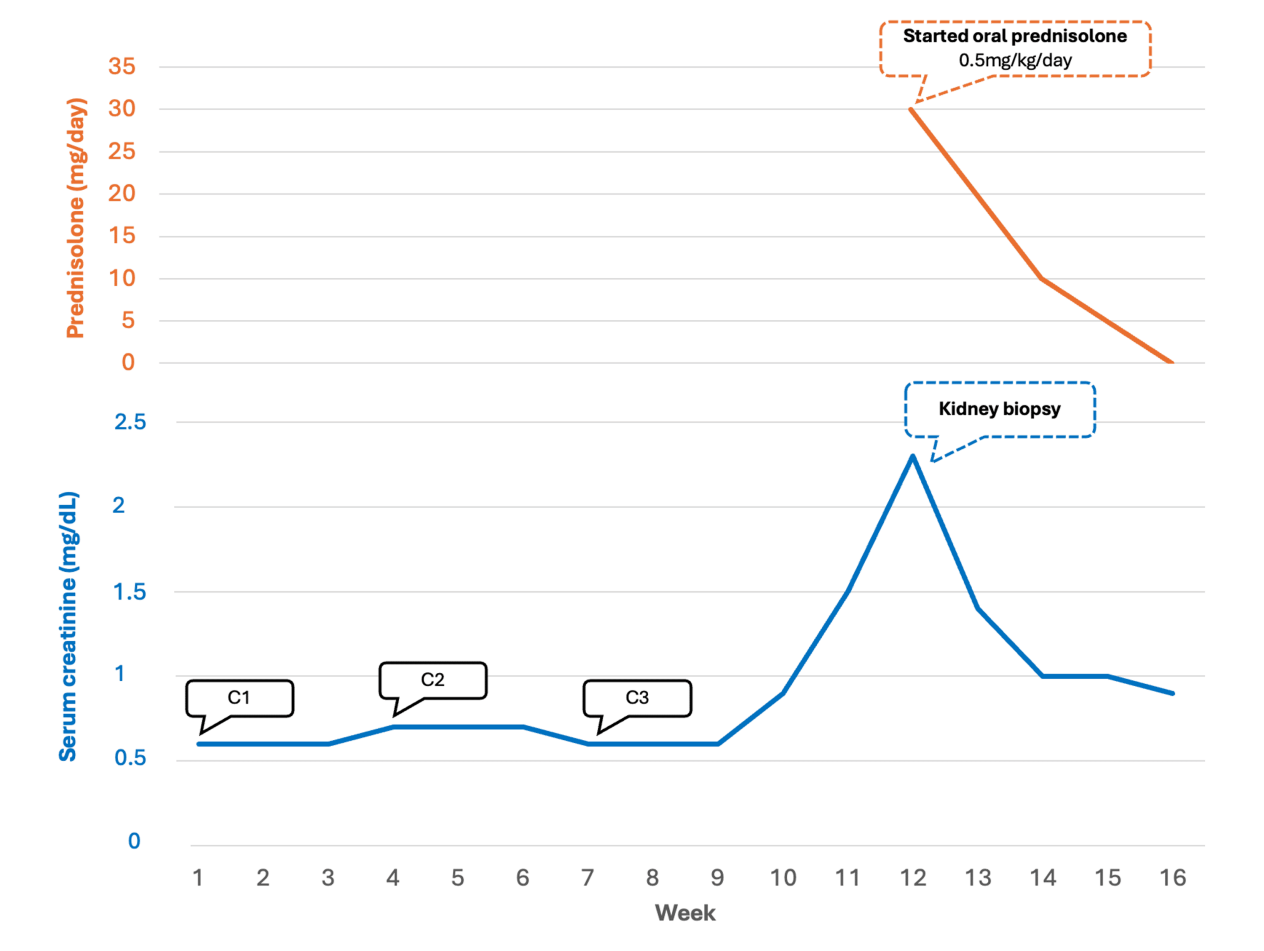
Patient’s renal function over time. C1-3: cycle 1-3 of neoadjuvant treatment (chemotherapy plus pembrolizumab).

Pembrolizumab was discontinued, and after four weeks of oral prednisolone therapy, the patient's renal function returned approximately to its baseline. Renal parameters remained stable following steroid withdrawal (sCr 0.9 mg/dL) (Figure 2).

## Discussion

ICIs are currently among the most frequently prescribed therapies for cancer. As the utilization of these agents continues to rise, the incidence of patients presenting with irAEs is expectedly increasing. The pathophysiology of irAEs remains unclear, but immune checkpoint blockade may cause inflammation in any organ by disrupting immunologic homeostasis [1].

Regarding renal irAEs, ICI-related AKI is a rare complication reported in 2-5% of patients treated with ICI [5,7,8]. In breast cancer, ICI-related AKI has been reported in <1% prospective trials [9]. ATIN represents the most common manifestation of ICI-related AKI [8,10]. Several factors may be associated with an increased risk of ICI-related AKI, including prior autoimmune condition, previous renal impairment, female gender, dual ICI therapy, and use of proton pump inhibitors or nonsteroidal anti-inflammatory drugs [9,10]. Nevertheless, data on ICI-related AKI are still limited and mainly come from small case series and oncological studies. A median time of nine months has been described between the start of therapy with pembrolizumab and the AKI onset, ranging from one to 16 months [11].

In cases of suspected ICI-induced nephritis, it is important to rule out other causes of renal failure, including hypovolemia, medication, obstruction, and infection. Urinalysis, including urine sediment examination and assessment of albumin-to-creatinine ratio (ACR) and polymerase chain reaction (PCR), is extremely valuable for its diagnosis. ICI therapy should be interrupted, as well as other nephrotoxic drugs, or permanently discontinued, depending on the severity of the renal insufficiency [10,11]. The European Society for Medical Oncology (ESMO) guidelines recommend withholding ICI and starting corticosteroids (0.5-1 mg/kg/day prednisolone equivalents, increasing to 1-2 mg/kg/day or IV methylprednisolone pulse doses of 250-500 mg for three days if the condition worsens) for patients with an sCr increase two to three times their baseline levels [12]. Once renal function improvement (sCr <1.5 times above baseline or baseline), steroid taper can be initiated over four to six weeks [10].

Due to its rarity, it is highly recommended that renal irAEs be managed through a multidisciplinary approach, with early consultation with nephrologists being essential. Renal biopsy plays a valuable role in confirming the diagnosis of ICI-related AKI. However, the decision to perform a biopsy should be individualized, considering the clinical context as well as the patient’s overall condition. Most importantly, the initiation of empiric corticosteroid therapy should not be delayed when there is a strong clinical suspicion of ICI-related AKI [10,13]. With prompt treatment initiation, most patients achieve complete or partial renal recovery. Evidence suggests that starting corticosteroid therapy within three days of ICI-related AKI onset greatly increases the likelihood of full kidney recovery [10].

Oncologic guidelines regarding ICI re-challenge in cases of ICI-induced nephritis vary, but it is generally recommended that the permanent discontinuation of ICI be considered if grade 3 toxicity or higher (according to Common Terminology Criteria for Adverse Events (CTCAE), version 5.0). For selected patients with limited therapeutic options, ICI rechallenge may be considered despite reported recurrence rates of ICI-related AKI of approximately 16-23% in observational cohorts. In this setting, secondary prophylaxis may include prednisolone 10 mg daily, particularly in those with severe initial ICI-related AKI or when rechallenge occurs within two to four months of the initial episode. Nevertheless, additional evidence is required to guide clinical decision-making [10-12].

With the approval use of pembrolizumab in early-stage triple-negative breast cancer, as well as other ICI agents now entering clinical trial testing in multiple malignancies, more ICI-related AKI is being reported, remaining a potentially serious complication that needs to be screened and monitored carefully in this population [9]. Further research is necessary in the areas of development and early detection of irAEs. Promising studies on noninvasive biomarkers are currently being conducted to improve the identification of patients for immunotherapy re-challenge [10].

## Conclusions

As ICI therapy becomes more widely used in cancer treatment, oncologists must be aware of possible side effects. Although rare, ICI-related AKI is a severe and potentially irreversible complication, requiring early recognition and prompt intervention. Treatment of ICI-induced ATIN includes holding ICI, discontinuing other ATIN-associated drugs, and starting corticosteroids as first-line therapy as soon as the suspicion is raised. Immediate corticosteroid therapy often results in either complete or partial renal function recovery. Multidisciplinary management is highly recommended, and kidney biopsy remains the gold standard for its diagnosis. Further research is required to improve the prevention, detection, and management of irAEs.
